# The effect of explantation on systemic disease symptoms and quality of life in patients with breast implant illness: a prospective cohort study

**DOI:** 10.1038/s41598-022-25300-4

**Published:** 2022-12-06

**Authors:** G. R. Bird, F. B. Niessen

**Affiliations:** 1 Department of Plastic, Reconstructive and Hand Surgery, Amsterdam University Medical Centre, Amsterdam, The Netherlands; 2 MITTSU Institute, Amsterdam, The Netherlands

**Keywords:** Outcomes research, Immunology

## Abstract

Silicone breast implants (SBIs) have been subject to scientific scrutiny since the 1960’s because of their potential link with systemic disease symptoms. Breast implant illness (BII) is a cluster of over 56 (systemic) symptoms attributed by patients to their SBIs. BII remains an unofficial medical diagnosis, although its symptoms include but are not limited to the clinical manifestations of autoimmune/inflammatory syndrome induced by adjuvants (ASIA). The aim of this study was to prospectively analyse the effect of explantation on clinical manifestations of ASIA/BII symptoms, as well as to compare (breast-surgery specific) QoL in patients pre- and postoperatively while recording relevant perioperative/patient data. A prospective cohort study was conducted on 140 patients consulting a single surgeon for explantation of SBIs at a single clinic from 2019 to 2021 via their general practitioner, a medical specialist or self-referral. Of all patients, medical (implant) history, lifestyle factors and biometric data were obtained. Patients filled out a novel ASIA/BII symptom-survey termed the ASIA-scale, three domains of the SF-36 and the augmentation module of the BREAST-Q before and four months after the operation. A total of 109 patients completed both the pre- and postoperative survey with a mean follow-up duration of 205 days. There was a significant decrease in all individual symptom scores as well as ASIA-scale summary scores after explantation (*p* < .001). All SF-36 subdomains showed significant improvement postoperatively (*p* < .001). The BREAST-Q subdomain ‘satisfaction with breasts’ improved significantly after explantation (*p* = .036). No statistically significant association was found between any clinical parameters (such as age, capsulectomy, rupture etc.) and the recovery of symptom scores. This is the largest prospective cohort study on SBI explantation to date showing significant improvement of the most common systemic complaints in SBI patients as well as improvement of satisfaction with breasts and overall quality of life.

## Introduction

Silicone breast implants (SBIs) have been subject to scientific scrutiny since the 1960’s because of their potential link with systemic disease symptoms^1–4^. Breast implant illness (BII) is a cluster of over 56 (systemic) symptoms attributed by patients to their SBIs^5–7^. BII remains an unofficial medical diagnosis, although its symptoms include but are not limited to the clinical manifestations of autoimmune/inflammatory syndrome induced by adjuvants (ASIA)^8,9^. The clinical manifestations of ASIA are: myalgia or myositis, arthralgia and/or arthritis, chronic fatigue, neurological manifestations, cognitive impairment and pyrexia^10^. Interestingly, the many symptoms of BII also have overlap with, or even fully meet, the diagnostic criteria of chronich fatigue syndrome (CFS)^11^, fibromyalgia^12^, sarcoid-like disease and undifferentiated connective tissue disease (CTD)^13,14^. This overlap with defined diseases, as well as the large amount of women ascribing systemic symptoms to their SBIs^5–7^, make BII an entity that should be studied.

### Disease management and study aim

The cornerstone of therapy for systemic symptoms in SBI patients appears to be explantation of SBIs, showing amelioration of symptoms in numerous studies^15–18^. Yet prospective studies are needed to evaluate the effect of explantation^9,19,20^. Clearly, further investigation is warranted into pre- and postoperative quality of life^21^, especially with the growing popularity of autologous fat grafting simultaneous with explantation^19,22^. Additionally, there is a need to record detailed patient history, lifestyle factors and relevant perioperative/patient data in explantation studies.

The aim of this study, therefore, was to prospectively analyse the effect of explantation on clinical manifestations of ASIA/BII symptoms, as well as to compare health-related and breast surgery-specific QoL in patients pre- and postoperatively while recording relevant perioperative/patient data. Secondary objectives were to evaluate the value of substitute cosmetic procedures such as autologous fat grafting on QoL and the effect several other clinical parameters on recovery of symptom scores.

## Method

A prospective cohort study was conducted on all patients (n = 140) who underwent explantation of SBIs by a single surgeon at a single clinic (MITTSU Institute, Amsterdam, The Netherlands) from January 2019 until September 2021. MITTSU Institute is a plastic surgery clinic that specializes in explantation of breast implants.

Patients were referred either by their general practitioner or a medical specialist after analysis for local and/or systemic complaints in relation to SBIs. Some came to the clinic by self-referral. All patients ascribing their symptoms to BII were considered for this study. We opted not to exclude those who opted for explantation for different reasons, resulting in a small control group. Hence, all patients opting for explantation after first consultation were included in this study.

At the clinic, detailed patient history was taken and a general assessment including physical examination was performed. Patient history (including implantation history) and current medication were obtained by anamnesis and via the referral letter of the general practitioner. Patients were interviewed by the surgeon with special attention given to implant related complaints, duration/onset of complaints, lifestyle factors (smoking, alcohol and drug abuse) and allergies. Physical examination consisted of inspection/palpation of the prostheses, registering the corresponding Baker score^23^ and calculating patients BMI. All patients signed an informed consent form before enrolling in the study.

### Surgical methods

Patients were consulted on explantation and additional subsequent surgical options such as autologous fat grafting and mastopexy. Patients who opted for autologous fat grafting and/or mastopexy did so using shared decision making. Patients were treated using a general sterile surgical technique. Explantation was performed using an existing or newly formed inframammary incision or through concurrent mastopexy if performed. Total or partial capsulectomy was performed in case of calcification or thickening of the periprosthetic capsule. In the case of silicone leakage resulting in silicone granuloma, the granuloma was completely excised. In the case of rupture, the surgical site was cleaned and disinfected using Hibicet® disinfectant. Implant exchange was not performed in the study group. If patients opted for autologous fat grafting, liposuction at various sites was performed, followed by mammary reintroduction via a layered technique using a blunt tip infiltration needle.

Numerous perioperative variables were recorded including: implant brand/size, occurrence of rupture (per side), abnormalities of the implant (disfiguration, aspect, disinfection), and if total or partial capsulectomy (per side), mastopexy or autologous fat grafting was performed.

### Questionnaires

Patients were asked to complete three questionnaires pre- and postoperatively. The preoperative questionnaires were sent after the patient first presented. The postoperative survey was sent four months after the operation. A general reminder was sent in September and December of 2021 to those who failed to respond at the first postoperative evaluation.

The first questionnaire, hereafter described as the ASIA-scale, was developed by the surgeon of MITTSU clinic. This novel questionnaire of 19 items consists of the clinical manifestations of ASIA^8^ as well as the most commonly reported BII symptoms, based on expert opinion (see Appendix). The symptom intensity was rated using a 5-point Likert scale ranging from 0 (I haven’t experienced this symptom at all) to 4 (I have severely experienced this symptom). The second questionnaire is a truncated version of the SF-36 focussing on the subdomains ‘general health’, ‘physical functioning’ and comparison of ‘change of symptoms one year ago’. The SF-36 (previously RAND-36) is a comprehensive short-form generic profile HRQoL measure. Its validity has been well established in numerous patient populations^24^. The third and final questionnaire, the BREAST-Q, is a validated breast surgery-specific patient-reported outcome measure^25^. Three sub-domains were selected for evaluation, using the augmentation module: ‘satisfaction with breasts’, ‘sexual well-being’ and ‘psychosocial well-being’. The electronically administered BREAST-Q yields highly reliable, clinically meaningful data for use in clinical outcomes research^26^. The questionnaires were administered online, using SurveyMonkey (Bain & Company, Inc., Fred Reichheld and Satmetrix Systems, Inc., 1999–2021) which was set to generate an anonymous respondent ID used for matching the pre- and postoperative response.

### Definitions

Patients who consulted for explantation for reasons other than BII or professionally established ASIA were termed ‘asymptomatic’, whereas those suffering from BII or ASIA were termed ‘symptomatic’. ‘Duration of silicon exposure’ in years was defined as the number of years from the first implantation to the year of explantation. ‘Duration of BII symptoms’ was defined as the number of years from the moment the patients first experienced symptoms attributed to BII until the explantation. ‘Time to the onset of BII symptoms’ was calculated by subtracting the duration of symptoms from the duration of silicon exposure. ‘Recovery of ASIA symptoms’ was the difference between pre- and postoperative ASIA-scale summary score. The same calculation was made for improvement of SF-36 and BREAST-Q subdomains. Patients who experienced symptoms > 5 years were defined as a group with ‘long duration of symptoms’, the remaining patients formed ‘short duration of symptoms’ group.

### Data analysis

The collected data were analysed using SPSS (IBM SPSS Statistics for Windows 27.0, USA, 1994–2021). A power analysis (paired-t, two sided)^27^ was performed prior to the study (parameters: α = 0·05, β = 0·2, SD = 20, σd = 18, *E* = ·28)^28^. A sample size of 104 was calculated, concluding that the actual sample size (n = 140) gives a satisfactory probability of giving a correct rejection of the null hypothesis.

For the evaluation of perioperative data, implant data and other characteristics, descriptive statistics of the patient cohort are presented. Continuous variables such as age were reported as mean and standard deviation (SD). Categorical variables such as rupture rate were reported as percentages. Questionnaire summary scores were converted into continuous variables (0–100 scores). In case of the BREAST-Q and SF-36, higher scores mean greater satisfaction or better QoL. Ordinal data such as individual survey questions were compared pre- and postoperatively using Wilcoxon signed-rank test. Continuous variables were compared pre- and postoperatively using a paired-t analysis. When comparing continuous variables between groups using an independent samples t-test, Levene’s test was performed to assess homogeneity of variances. Uni- and multivariate associations (crude and adjusted) between various patient characteristics, (peri)operative and implant-related variables were estimated using Pearson’s (or Point-Biserial) correlation and linear regression coefficients for their impact on questionnaire outcomes. A significance level of *p* ≤ 0·05 was deemed statistically significant.

### Ethics committee approval

All patients signed informed consent forms prior to enrolling in this study. Consent to publish was also obtained. Therefore, the study was approved by the METC VUMc (Ethics committee of VUMc, Amsterdam UMC). In our study we adhered to local guidelines and regulations regarding ethical care or research and consent from patients accordingly.

## Results

A total of 140 patients underwent explantation surgery (without reimplantation) of SBIs between the beginning of 2019 and the end of 2021. Thirty-one patients (22%) did not respond to the postoperative questionnaires despite repeated requests. The postoperative survey was completed within a range of 88–670 days after explantation, with a mean value of 205 days.

Demographic and clinical characteristics of the study group, categorized by organ system or symptom group, are described in Table 1.

**Table 1:** Demographic and clinical characteristics of study group (n = 140).

Characteristic	Value	Percentage†
Age at time of surgery, mean ± SD in years (range)	43.2 ± 10.6 (24–69)	NA
**Medical history**		
Autoimmune, rheumatologic or connective tissue disease*	11	7.9%
Other immune mediated diseases**	4	2.9%
Functional somatic syndromes***	7	5.0%
Thyroid disease♣	11	7.9%
Cardiovascular disease♣♣	9	6.4%
Respiratory disease♣♣♣	11	7.9%
Gastrointestinal disease*♣	9	6.4%
Skin disease**♣	6	4.3%
Musculoskeletal disease*♣♣	7	5.0%
Malignancy♣♣*	6	4.3%
Psychiatric/psychological disorders♦	14	10.0%
Headaches♦♦	8	5.7%
Other♦♦♦	11	7.9%
Medication		
Corticosteroids	3	2.1%
Antihistamines	7	5.0%
Analgesics	8	5.7%
Biologicals	2	1.4%
Levothyroxine	8	5.7%
Statins	2	1.4%
Inhalers	9	6.4%
Proton pump inhibitors	8	5.7%
Tryptans	5	3.6%
Antidepressants	14	10.0%
Benzodiazepines	8	5.7%
Others°	15	11.7%
Allergies	57	40.7%
**Lifestyle factors / intoxications**		
Alcohol (mild; 0–1 unit/day)	32	22.9%
Alcohol (severe; 1–2 or more /day)	7	5%
Smoking	12	8.6%
Drugs (at least monthly)	3	2.1%

*SLE, Sarcoidosis, Rheumatoid arthritis, Raynaud’s phenomenon, Lichen planus, Lichen sclerosus, Ehlers-Danlos, Vitiligo. **IgA deficiency, ITP, Still's disease, polyclonal IgM Syndrome. ***CFS, FM. ♣Hypo/hyperthyroidism. ♣♣Hypertension, Hypercholesterolaemia, Palpitations. ♣♣♣Asthma, COPD, rhinitis, sinusitis. *♣IBS, Ulcerating colitis, GERD. **♣Eczema, psoriasis, Lichen planus, Lichen sclerosus. *♣♣Arthritis, Disc herniation. ♣♣* Of which 2 breastcancer♦Depression, Burnout, ADHD, anorexia/bulimia. ♦♦Migraine, Tension headaches.♦♦♦Insomnia, CVA, braincyst, Lyme's disease, adverse reaction to dermatologic filler, Poland syndrome, Osteoporosis, polyneuropathy. °Of which 5 hormone replacement therapy and 1 methotrexate. †Percentage over total SBI population (n = 140). NA = Not applicable. Missing values for medication = 3, allergies = 7, lifestyle factors = 9.

Seventeen patients (12·1%) had a previous medical record of immune mediated disease. Six patients (4·3%) had previously been diagnosed with fibromyalgia and one patient (0·7%) had pre-existing chronic fatigue syndrome (CFS). Five patients (3·6%) had Irritable Bowel Syndrome (IBS). A total of six patients (4·3%) had a prescription for immune modulating medication (corticosteroids, biologicals or methotrexate). A total of twenty-two patients (15·7%) chronically used psychoactive medication (i.e. antidepressants and benzodiazepines). Fifty seven patients (40·7%) were allergic to one or more substances. The three most occurring allergies were antibiotics (17%), specific foods/nuts (17%) and various types of pollen (15%).

Findings during physical examination and implant specific history are separately reported in Tables 2 and 3.

**Table 2:** Findings during physical examination (n = 140).

Finding	Value		Percentage*	
BMI, mean ± SD (range)	22.4 ± 2.9 (17.5–30.8)		NA	
Underweight (BMI < 20)	25		17.9%	
Overweight (BMI > 25)	25		17.9%	
Obese (BMI > 30)	4		2.7%	
Capsule type	Left	Right	Left	Right
Baker score 1	99	91	70.7%	65%
Baker score 2	14	23	10%	16.4%
Baker score 3	3	6	2.1%	4.3%
Baker score 4	21	17	15%	12.1%

*Percentage over total SBI population (n = 140). NA = Not applicable. Missing values for capsule type = 17L, 3R; BMI = 7.

**Table 3:** Implant specific history (n = 140).

Characteristic	Value	Percentage*
Duration of silicone exposure, mean ± SD in years (range)	14.4 ± 7.4 (1–46)	NA
Duration of BII symptoms, mean ± SD in years (range)	6.2 ± 5.5 (0–26)	NA
Time to onset of symptoms, mean ± SD in years (range)	7.0 ± 7.4 (0–30)	NA
Reimplantation	41	29.3%
Once	32	22.9%
Multiple	9	6.4%
Implant size, mean ± SD in cc’s (range)**†**	300.6 ± 76.1 (150–570)	NA
Reason for implantation		
Cosmetic	137	97.9%
Reconstructive	3	2.1%
Implant type		
Silicone	134	95.7%
Saline	6	4.3%
Implant brand	Allergan (31.8%), Mentor (16.2%), Eurosilicone (14.9%)	

*Percentage over total SBI population (n = 140). NA = Not applicable. **†** 12 missing values.

Thirty-eight patients (25·7%) had capsular contracture (i.e. Baker 3 or 4; firm, deformed breast) of at least one breast. Only two patients had SBIs implanted for reconstructive purposes after breast cancer and one patient because of Poland syndrome^29^. Thirteen patients (9·3%) reported having no BII symptoms when consulting for explantation, and were termed asymptomatic. Of those thirteen, nine explanted for cosmetic reasons and four because of local complaints (of which two had MRI confirmed rupture, without systemic symptoms).

In forty-three patients (30·7%), the surgeon deemed it necessary to perform a capsulectomy (see Table 4). The majority of patients had an additional cosmetic procedure performed (92·8%), either mastopexy or autologous fat reconstruction.

**Table 4:** Operative details and findings (n = 140).

Finding/procedure	Value		Percentage*	
Rupture rate a priori	20		14.2%	
Right	10		7.1%	
Left	5		3.6%	
Bilaterally	5		3.6%	
**Implant abnormalities**				
Discoloration†	18		12.9%	
Excess fluid surrounding implant	6		4.3%	
Excess scar tissue/calcification	4		2.8%	
Intraoperative rupture	3		2.1%	
Siliconoma	2		1.4%	
Decrease in volume > 50 cc’s	2		1.4%	
Implant malposition/rotation	2		1.4%	
Capsulectomy	Left	Right	Left	Right
Partial	21	22	15%	15.7%
Total	10	11	7.1%	7.9%
Autologous fat reconstruction	119		85%	
Mastopexy	11		7.9%	

*Percentage over total SBI population (n = 140). †17 of yellow aspect, 1 brownish discoloration. Total number of missing values = 0.

### Primary outcome: effect of explantation

As can be seen in Table 5, the ASIA-scale summary score decreased significantly (i.e. condition improved) after explantation. For the asymptomatic patients who completed both pre- and postoperative surveys (n = 11), the mean score decreased from 28·3 to 21·6 after explantation (*p* = ·121). In the group of symptomatic patients (n = 98) the mean score decreased from 52·0 to 32·7 (*p* < ·001). The difference in reduction of ASIA summary scores between the symptomatic and asymptomatic group was statistically significant (*p* = ·012, equal variances not assumed).

**Table 5:** Comparison of pre- vs. postoperative mean questionnaire (subdomain) summary scores (n = 109).

Questionnaire	Preoperative (± SD)	Postoperative (± SD)	Mean difference (SE)	*P* value†
ASIA	49.5 (± 17.9)	31.6 (± 17.2)	17.9 (1.6)	< .001
Satisfaction with breasts*	57.9 (± 16.4)	62.8 (± 21.7)	4.9 (2.5)	.036
Sexual wellbeing*	59.7 (± 21.5)	59.5 (± 21.7)	0.2 (2.3)	.771
Psychosocial wellbeing*	59.8 (± 15.4)	62.3 (± 17.4)	2.5 (1.6)	.121
General health♣	36.2 (± 22.2)	48.3 (± 24.1)	12.1 (2.2)	< .001
Change vs. 1 year ago♣	36.3 (± 24.4)	71.6 (± 25.4)	35.3 (3.4)	< .001
Physical health♣	76.9 (± 23)	85 (± 23.2)	8.1 (2)	< .001

^**†**^Paired samples t-test. *BREAST-Q subdomains: higher score indicates better QoL. ♣SF-36 subdomains: higher score indicates better QoL.

**Figure 1:**
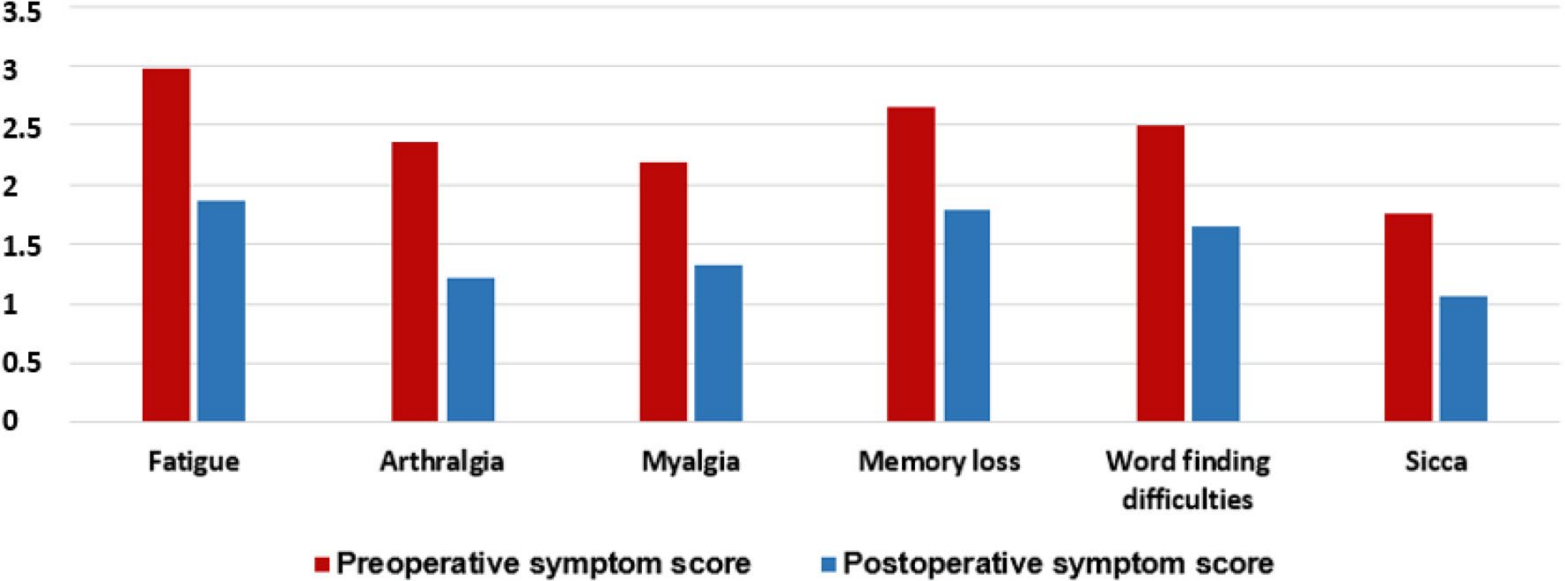
Comparison of preoperative vs. postoperative ASIA major criteria clinical manifestations mean symptom scores in the symptomatic group (n = 98) using Wilcoxon Signed-Ranks test (*p* < .001).

The ASIA questionnaire symptoms that were most frequently reported as a high score preoperatively (i.e. mean Likert score > 2 out of 4) were: fatigue (2·88), arthralgia (2·26), myalgia (2·06), memory loss (2·58), word finding difficulties (2·41), short tempered (2·21), back/neck pain (2·73), cold hands/fingers (2·33) and brain fog (2·31). No symptoms in the asymptomatic group scored a mean of > 2 before surgery. Furthermore, there was a significant decrease in all 19 individual ASIA questionnaire symptom scores when comparing pre- and postoperative data (*p* < ·001). Postoperative improvement of the ASIA-syndrome major criteria clinical manifestations for symptomatic patients, is shown in Fig. 1.

Additionally, all SF-36 subdomain summary scores (‘general health’, ‘change from one year ago’ and ‘physical health’) increased significantly (i.e. QoL improved) from preoperative to postoperative. Of the BREAST-Q subdomains, only ‘satisfaction with breasts’ showed an increase after surgery (*p* = 0.0.36, Table 5). There were no differences in improvement after surgery in all SF-36 and BREAST-Q subdomains between symptomatic and asymptomatic patients.

### Secondary outcome: variables with effect on recovery

Several variables where evaluated for their effect on the recovery of symptoms, as is outlined in Table 6. There was no significant association between the performance of a capsulectomy (either partial or total) and recovery of ASIA-scale symptoms or improvement of general health (*p* = ·246) and physical health (*p* = ·972). There was also no difference between patients with an established autoimmune, rheumatologic or connective tissue disease or a functional somatic syndrome and patients without such illness, in recovery of symptoms (*p* = ·470). Yet, the postoperative ASIA-scale summary scores and SF-36 subdomain ‘general health’ scores were significantly higher (*p* = ·022) and lower (*p* = ·017) respectively in patients with these preexisting illnesses. Patients with a long duration of BII symptoms (i.e. > 5 years) had better recovery scores (*p* = ·017, equal variances assumed), while their preoperative ASIA-scale scores did not differ significantly compared with patients who experienced BII symptoms for less than 5 years (*p* = ·148).

**Table 6:** Pearson’s or Point Biserial correlation and univariate linear regression coefficients of variables possibly affecting symptom recovery in relation to recovery of ASIA summary score*.

Independent	Crude			
	R	B	CI	*P* value
Age	−.110	−.169	−.48 to .15	.289
BMI	.079	.418	−.67 to 1.50	.447
Intoxications	−.112	−3.75	−10.2 to 2.75	.255
Allergies	.176	5.79	−.529 to 12.1	.072
IMD/FSS group†	.071	2.78	−4.83 to 10.4	.470
Implant rupture (before or during operation)	.032	1.47	−7.54 to 10.5	.747
Implant size	.046	.009	−.031 to .050	.649
Capsular contracture on either side	−.138	−4.79	−11.5 to 1.94	.161
Duration of silicon exposure	−.010	−.020	−.422 to .383	.923
Time to onset of BII symptoms	−.128	−.248	−650 to .153	.223
Capsulectomy (partial or total)	−.061	−2.10	−8.78 to 4.58	.543

*For population having completed both questionnaires (n = 109). R = correlation. B = regression coefficient. CI = confidence interval. †i.e. SLE, Sarcoidosis, Rheumatoid arthritis, Raynaud’s phenomenon, Lichen planus, Lichen sclerosus, Ehlers−Danlos, Vitiligo, IgA deficiency, ITP, Still's disease, polyclonal IgM Syndrome, fibromyalgia, CFS, IBS. Dependent variable = recovery of ASIA-scale scores. R = correlation. B = regression coefficient. CI = confidence interval.ftab.

The improvement of QoL questionnaires was also found to have no association with performance of a capsulectomy, preexisting IMD/FSS, implant rupture/size, capsular contracture, time to onset of BII symptoms and duration of silicon exposure.

The duration of the follow up period was not significantly associated with either recovery of symptoms (*p* = ·874) or improvement of any QoL.

## Discussion

The aim of this prospective, cohort study was the evaluation of the effect of explantation on systemic complaints in SBI patients, as well as the comparison of health-related and breast-surgery-specific quality of life, before and after the operation. Various clinical and surgical parameters were also assessed for their effect on the recovery of symptoms or improvement of quality of life.

It was found that all common BII symptoms reduced significantly after explantation. Others have retrospectively reviewed the effect of explantation^17,18,30^, but the potential influence of comorbidities was not always considered^16^. One previous study by Lee et al. did prospectively evaluate the effect of explantation compared with controls, with slightly shorter follow up and elaborate capsule analysis^9^. Despite differences in design and limitations, these studies report similar findings to this paper, showing amelioration of all common systemic symptoms in SBI patients.

These results can be explained by the main theory of BII pathophysiology: the immunological hypothesis, where SBIs are the chronic stimulus that causes overreaction of the immune system^8,14,31^. A similar overactive and sustained response, in the form of the foreign body reaction, is seen in capsular contracture^31^. This could explain why explantation leads to relatively swift reduction of all systemic symptoms, in this study with a mean recovery period of 205 days.

Yet a residual level of symptoms remains after explantation (33/100), indicating an ongoing or fading disease process. Although there was a significant difference in recovery of symptom scores between symptomatic and asymptomatic patients, it should be noted that asymptomatic patients also had a residual symptom score (22/100). This is comparable to the findings of Colaris and Cohen Tervaert, suggesting that a particular group of SBI patients develop more severe complaints than the remaining SBI population^33^, which could be indicative of a psychological component^14^. This is in line with the current study, where memory loss, brain fog and word finding difficulties are among the highest rated symptoms. Moreover, Misere et al., have even found no significant difference in ASIA symptoms in women with SBIs compared to the general population^21^.

Furthermore, health-related quality of life improved across all subdomains. Misere et al. have compared health-related quality of life in SBI patients and healthy controls, finding lower QoL in women with SBIs^21^. Yet to our knowledge, validated QoL questionnaires have not yet been used to evaluate the effect of explantation.

Additionally, satisfaction with breasts improved significantly after explantation. This could potentially be due to the performance of additional cosmetic procedures such as mastopexy and autologous fat grafting. Autologous fat grafting has been previously studied directly after explantation, finding satisfaction in over 80% of patients. However, outcomes were evaluated with non-validated questionnaires^34^.

Interestingly, the results of this study could not identify any clinical parameters with a significant influence on recovery of symptoms. Even adjusted for age, BMI and smoking no significant association could be identified. This is at variance with the paper by Wee et al. which reported a more significant improvement of symptoms in patients with capsular contracture and obesity^16^. Wee et al. argue that the pro-inflammatory state of obesity and capsular contracture can contribute to the hyperactive immune response, and thus lead to better improvement after surgery. Notably, in the present cohort, only 16·4% had contracture, compared with 55·4% in Wee’s study, and, only four patients qualified as obese, potentially explaining the difference in results. Other explanations might be the unreliability of the Baker score^23^ or the fact that no adjustment was made for potential confounders in the retrospective study.

Still, it was found that postoperative symptom scores were significantly higher in patients with a previous history of immune mediated disease or a functional somatic syndrome. It has previously been reported that patients with autoimmune diagnoses improve less after explantation^9^.

Moreover, the fact that performance of a capsulectomy did not significantly influence the recovery of symptoms could indicate that the immunological hypothesis is not yet fully understood. It is possible that the foreign body reaction and loco-regional silicone migration are just one aspect of the problem.

One could further argue that social media influences^5–7^, next to psychological predisposition^20,33^ as already mentioned above, play a significant role in the eruption and severity of systemic symptoms in SBI patients. Interestingly, the duration of follow-up did not affect the recovery of symptoms, whereas a greater improvement would be expected after a longer follow-up period. Others even report no improvement of recovery from 30 days onward postoperatively^16^, raising the question as to whether homeostasis could have occurred so soon. Could psychological factors be of influence here? This could be the reason why only certain individuals report complete remission of symptoms.

A combination of the immunological and psychological hypotheses might lead to the development of additional treatment after surgery, leading to improved remission of symptoms in patients. Indeed a multifaceted approach to BII research, based on a clinical, external and psychological aspects, could fill the gaps in current knowledge of BII aetiology. Future efforts could focus on the interplay between psychological and immunological factors.

## Limitations

The primary limitation of our study is the possibility of sampling bias, impacting the validity of the study. The majority of patients consulting MITTSU clinic for explantation were referred with ASIA or BII complaints. This raises the question whether the associated data are an accurate representation of the SBI population as a whole, as has been reported before^21^. Although, it is needless to say that sampling bias does not affect the results of this study for those actually reporting to have BII. Interestingly, the possibility of sampling bias supports the notion that a selected group of SBI patients experience more severe systemic symptoms, as already mentioned above.

## Conclusion

This is the largest prospective cohort study on SBI explantation to date showing significant improvement of the most common systemic complaints in SBI patients as well as improvement of overall quality of life and satisfaction with breasts. Clinical parameters influencing the recovery of symptoms or improvement of quality of life could not be identified. Future research should focus on the immunological basis of breast implant illness possibly combined with a psychological assessment in order to provide additional treatment after explantation, to improve the level of residual symptoms with a prospect of complete remission.

## Data Availability

The datasets used and/or analysed during the current study available from the corresponding author on reasonable request. However, the dataset is still under review for analysis for current and soon to be published work.

## Instruction for the use and scoring of the novel ASIA-scale symptom survey

- **Scoring of 5 point Likert-scale:**
  1. 0 = I haven’t experienced this symptom at all
  2. 1 = I have rarely experienced this symptom
  3. 2 = I sometimes experience this symptom
  4. 3 = I have regularly experienced this symptom
  5. 4 = I have severely/constantly experienced this symptom
- **Conversion to summary (continuous) scores:**
  1. Convert Likert-scale scores to continuous scores per individual question (1 to 0, 2 to 25, 3 to 50 etc.)
  2. Average scores of symptoms most accurately resembling ASIA syndrome symptoms and multiply by 0.5
  3. Average scores of remaining symptoms and multiply by 0.5
  4. Add scores of steps 2 and 3 together to calculate ASIA-scale summary score

See Table

**Table 7:** Novel ASIA-scale symptom survey and respective weight for summary score.

ASIA major criteria symptoms (50% of total score)	Additional BII symptoms (50% of total score)
Fatigue	Night sweats
	Feelings of depression
Myalgia	Chest pain/discomfort
	Intolerance to specific food items
Arthralgia	Newly developed allergies
	Being short-tempered
Memory loss	Alopecia
	Skin abnormalities
Word finding difficulties	Back/neck pain
	Fungal infections
Sicca/Pyrexia	Cold hands/fingers
	Thyroid disease
	Brain fog

7.
